# Supplementary material for: Prevalence of hypertension, and related factors among adults in Wolaita, southern Ethiopia: A community-based cross-sectional study
Source: PLoS One. 2021 Dec 15;16(12):e0260403. doi: 10.1371/journal.pone.0260403 (PMC8673671; doi:10.1371/journal.pone.0260403)
Supplement: Attachment — Submitted filename: Responses to Reviewers.docx [file pone.0260403.s006.docx]

Dear Dr. Wali Khan,

We would like to thank you and the reviewers for your suggestions, and comments. We have worked on the comments provided by the reviewer. We have uploaded the following items during our resubmission: responses to the reviewers, Revised Manuscript with Track Changes, and Revised Paper without Track Changes (Manuscript). Please see below our one-by-one response and explanations to questions and comments. Our responses and explanations to the questions and comments are indicated in bullet points.

Journal Requirements:

Please review your reference list to ensure that it is complete and correct. If you have cited papers that have been retracted, please include the rationale for doing so in the manuscript text, or remove these references and replace them with relevant current references. Any changes to the reference list should be mentioned in the rebuttal letter that accompanies your revised manuscript. If you need to cite a retracted article, indicate the article’s retracted status in the References list and also include a citation and full reference for the retraction notice.

- Thank you so much, we have carefully reviewed the reference list, and all are written according to the PLOS ONE requirement.

  **Additional Editor Comments (if provided):**
  Dear authors/editorial staff members/Editor in chief!
  I would like to inform you that I have reached my decision that the manuscript numbered cited in the subject above is based on scientific background and fulfil and satisfy the standard of PLOS ONE for publication. I have checked out the points of reviewers raised during revision and responses given by the author(s) against each point. I am satisfied by the response of the author(s) for the points raised. As according to the reviewers comments objectives were not similar to the title and thus the findings were not related to the objectives and lack of consistency in the paper but this is now addressed by the authors accordingly. As Academic editor of this manuscript I decided to publish this submission in PLOS ONE.
- Thank you so much for such an encouraging response.

  **Reviewers' comments: Reviewer's Responses to Questions**

**Comments to the Author**
1. If the authors have adequately addressed your comments raised in a previous round of review and you feel that this manuscript is now acceptable for publication, you may indicate that here to bypass the “Comments to the Author” section, enter your conflict of interest statement in the “Confidential to Editor” section, and submit your "Accept" recommendation.

Reviewer #1: All comments have been addressed

Reviewer #2: All comments have been addressed

- Thank you so much.

2. Is the manuscript technically sound, and do the data support the conclusions?

The manuscript must describe a technically sound piece of scientific research with data that supports the conclusions. Experiments must have been conducted rigorously, with appropriate controls, replication, and sample sizes. The conclusions must be drawn appropriately based on the data presented.

Reviewer #1: Yes

Reviewer #2: Yes

- Thank you so much.

3. Has the statistical analysis been performed appropriately and rigorously?

Reviewer #1: Yes

Reviewer #2: I Don't Know

- Thank you so much.

4. Have the authors made all data underlying the findings in their manuscript fully available?

The [PLOS Data policy](http://www.plosone.org/static/policies.action#sharing) requires authors to make all data underlying the findings described in their manuscript fully available without restriction, with rare exception (please refer to the Data Availability Statement in the manuscript PDF file). The data should be provided as part of the manuscript or its supporting information, or deposited to a public repository. For example, in addition to summary statistics, the data points behind means, medians and variance measures should be available. If there are restrictions on publicly sharing data—e.g. participant privacy or use of data from a third party—those must be specified.

Reviewer #1: Yes

Reviewer #2: No

- Thank you for the comment; as we have responded previously, we will deposit the data to a public repository on the acceptance of the manuscript for publication.

5. Is the manuscript presented in an intelligible fashion and written in standard English?

PLOS ONE does not copyedit accepted manuscripts, so the language in submitted articles must be clear, correct, and unambiguous. Any typographical or grammatical errors should be corrected at revision, so please note any specific errors here.

Reviewer #1: Yes

Reviewer #2: Yes

- Thank you so much.

6. Review Comments to the Author

Please use the space provided to explain your answers to the questions above. You may also include additional comments for the author, including concerns about dual publication, research ethics, or publication ethics. (Please upload your review as an attachment if it exceeds 20,000 characters)

**Reviewer #1:** I suggest to accept it in the current form.
Authors succeeded to response to all of the raised points

- Thank you so much.

**Reviewer #2: Minor comment**
**Result**
Line 371---mean(SD) it is good if you write like 130.5 ±0.4

- We appreciate the comment, however, the "mean (SD)" is written in accordance with the guideline of PLoS ONE which states: "Properties of distribution. It should be clear from the text which measures of variance (standard deviation, standard error of the mean, confidence interval) and central tendency (mean, median) are being presented."

**Discussion**
Line 476-478---Why you raised this issue? Because as you told us this was not assed in your study. So it is good if you delete it.

- Thank you, we have deleted it.
  **Strength**
  Line 509---judgment of causality?? Your study design is cross-sectional how it could be???
  As my perception it is good you minimize or summarize it and make your strength & limitation to one paragraph
- We also accepted this comment and minimized the size.
  **Conclusion**
  Line 536—you write modifiable risk factors but it is good if you indicate this statement above (line 529) when you listing factors associated with blood pressure. Because this is conclusion so it is good if you show us those risk factors you identified in your study as modifiable and non-modifiable.
- Thank you, we accepted your comment and moved the statement to the place where you suggested, and listed modifiable risk factors.
  **Reference**
  Line 596—reference 10—randomly I checked this reference and for this article there is authors so, it is good if you see and revise it.
- Thank you so much for the comment, we have revised all the references and made corrections accordingly.

7. PLOS authors have the option to publish the peer review history of their article ([what does this mean?](https://journals.plos.org/plosone/s/editorial-and-peer-review-process#loc-peer-review-history)). If published, this will include your full peer review and any attached files.
If you choose “no”, your identity will remain anonymous but your review may still be made public.

**Do you want your identity to be public for this peer review?** For information about this choice, including consent withdrawal, please see our [Privacy Policy](https://www.plos.org/privacy-policy).

Reviewer #1: **Yes:** Ishag Adam

Reviewer #2: No

- No
