# Supplementary material for: Prevalence of hypertension, and related factors among adults in Wolaita, southern Ethiopia: A community-based cross-sectional study
Source: PLoS One. 2021 Dec 15;16(12):e0260403. doi: 10.1371/journal.pone.0260403 (PMC8673671; doi:10.1371/journal.pone.0260403)
Supplement: Attachment — Submitted filename: Responses to reviewers_recent.docx [file pone.0260403.s007.docx]

Dear Dr. Rosely Sichieri,

We would like to thank you for your suggestions, and comments. We have worked on the comments provided by you. We have uploaded the following items during our resubmission: Responses to the editor, Revised Manuscript with Track Changes, and Revised Paper without Track Changes (Manuscript). Please see below our one-by-one response and explanations to questions and comments. Our responses and explanations to the questions and comments are indicated in bullet points.

Thank you for submitting your manuscript to PLOS ONE. After careful consideration, we feel that it has merit but does not fully meet PLOS ONE’s publication criteria as it currently stands. Therefore, we invite you to submit a revised version of the manuscript that addresses the points raised during the review process.

You please should make all the changes in the tables and analysis suggested.

- Thank you so much, we have made the changes in the tables and analysis based upon your suggestion.

Please ensure that your decision is justified on PLOS ONE’s publication criteria and not, for example, on novelty or perceived impact.

- Thanks, we made our decisions based upon PLOS ONE's publication criteria.

If you would like to make changes to your financial disclosure, please include your updated statement in your cover letter. Guidelines for resubmitting your figure files are available below the reviewer comments at the end of this letter.

a. Please clarify the sources of funding (financial or material support) for your study. List the grants or organizations that supported your study, including funding received from your institution.

- South Ethiopia Network of Universities in Public Health (SENUPH) funded the research; which in turn is funded by the Norwegian Program for Capacity Development in Higher Education and Research for Development (NORHED), the grant number of this research was ETH-13-0025.
- Wolaita Sodo University provided logistics and technical support.

b. State what role the funders took on the study. If the funders had no role in your study, please state: “

- The funders had no role in study design, data collection, and analysis, decision to publish, or preparation of the manuscript.

c. If any authors received a salary from any of your funders, please state which authors and which funders.

- There is no author who received a salary from South Ethiopia Network of Universities in Public Health (SENUPH) or Norwegian Program for Capacity Development in Higher Education and Research for Development (NORHED). The principal author is the academic staff of Wolaita Sodo University, Ethiopia. Therefore, I receive a salary from the Wolaita Sodo University, Ethiopia. However, the university had no role in the study design, data collection and analysis, decision to publish, or preparation of the manuscript. Currently, I am on study leave for a PhD study. I am doing my PhD study in a joint PhD program at Hawassa University and the University of Bergen. And this study is part of my PhD work, where the Wolaita Sodo University has no direct role in this study.

d. If you did not receive any funding for this study, please state: I received funding and the financial information was given above.

**Additional Editor Comments (if provided):**

I am sorry to say that my understanding is that this revised version still requires many changes.
The paper is too long, and it lacks focus.

- Thank you, we appreciate your point and worked based on your comment.

1- There is no need of looking at levels of blood pressure and prevalence. Authors should keep only prevalence analysis. All diastolic and systolic analyses should be deleted.

- Thanks, we worked according to your suggestion. Therefore, we deleted diastolic and systolic analyses and analyzed only the prevalence of hypertension.

2- Title should be changed - prevalence of hypertension in adults of xxxx and related factors.

- We would also like to appreciate your comment, we have now revised the title, and wrote based upon your suggestion.

3- The aim as written is to compare urban and rural areas??? This is not true.
The aim is to determine the prevalence of hypertension and prehypertension and related factors.

- Thank you, we have also restated the aim of the study, according to your suggestion.

4- Multilevel analysis should be explained/ Was it used to account for the complex design? Are weighting included??? The ICC data is not necessary to show the intracluster? Complex surveys have specific programs called survey in SAS and Stata. They should be used.

- We accepted the comment and carried out the multivariate analysis using survey data analysis in STATA meaning after declaring the data set as survey data.

5- Keep only the factors related to hypertension in the introduction and adjust for those factors that make sense. Multivariate analysis adjusting all for all makes no sense.

- Thank you, in the introduction, we listed factors with public health importance from studies conducted in Ethiopia and elsewhere that showed association with hypertension, and from a practical point of view, we kept or adjusted for those factors in the multivariate analysis.

6- Tables are wrong. Prevalences in the new tables by each factor should be showed. Not percentages in the column.

- Thank you, we accepted the comment and worked accordingly.

7- No need of table 1. Make the first the prevalence of hypertension and prehypertension by characteristics of the population. Weighted prevalence.

- Thank you, we made the first table prevalence of hypertension and prehypertension by characteristics of the population as you suggested, and also carried out the weighted prevalence.

8- The same for tables 2 and 3. All should be combined in one also with the prevalence by factors. Excluded all lipids and family store.

- Thank you, we also made the second table as suggested above with the prevalence by the behavioral and other factors, excluding lipids and family history.

9- No need of table 4 and 5.

- Thank you, we have omitted tables 4 and 5. We have now only two descriptive tables.

10- Review all the results and discussion after new analysis

- We also appreciate all your comments. We wrote the results and discussion based upon the new analysis.
